# The Analgesic Properties of *Corydalis yanhusuo*

**DOI:** 10.3390/molecules26247498

**Published:** 2021-12-10

**Authors:** Lamees Alhassen, Travis Dabbous, Allyssa Ha, Leon Hoang Lam Dang, Olivier Civelli

**Affiliations:** 1 Department of Pharmaceutical Sciences, School of Pharmacy, University of California-Irvine, Irvine, CA 92697, USA; lalhasse@uci.edu (L.A.); tdabbous@uci.edu (T.D.); allyssch@uci.edu (A.H.); leonhd@uci.edu (L.H.L.D.); 2 Department of Developmental and Cell Biology, School of Biological Sciences, University of California-Irvine, Irvine, CA 92697, USA

**Keywords:** *Corydalis yanhusuo*, alkaloids, analgesia, pain, inflammation

## Abstract

*Corydalis yanhusuo* extract (YHS) has been used for centuries across Asia for pain relief. The extract is made up of more than 160 compounds and has been identified as alkaloids, organic acids, volatile oils, amino acids, alcohols, and sugars. However, the most crucial biological active constituents of YHS are alkaloids; more than 80 have been isolated and identified. This review paper aims to provide a comprehensive review of the phytochemical and pharmacological effects of these alkaloids that have significant ties to analgesia.

## 1. Introduction

Pain is described as a sensation that is felt as a result of a physically-hurting stimulus [1]. Pain can be divided into nociceptive, inflammatory, and neuropathic pain [1]. Nociceptive pain is defined as a form of physical pain that is experienced during external injury [2]. Inflammatory pain is classified as tissue damage and infiltration of immune cells, while neuropathic pain is described as pain that is experienced through any damage to the nervous system [2]. The transmission of pain sensation is relayed through affected neurons to the spinal cord, which then transfers the signal to the brain for processing [2]. To date, antinociceptive and anti-inflammatory drugs are the gold standard to manage pain, while anticonvulsants and antidepressants are used to treat neuropathic pain [3].

The CDC recommends anti-inflammatory drugs (i.e., nonsteroidal anti-inflammatory drugs known as NSAIDS and COX-2 selective inhibitors) to treat low to moderate types of pain [4]. However, for more severe pain, opiate drugs are the gold standard [4]. Opiates are shown to be effective for 70–80% of patients, hence they are the go-to when it comes to analgesia. However, these potent opiates cause a wide array of adverse side effects, such as tolerance, dependence, respiratory depression at high doses, and reduction in GI motility [5].

Pain itself has caused a huge burden in our healthcare system, as many patients suffer from adverse pain sensations, which ultimately reduces their quality of life [6]. The CDC reports that pain affects more than 50 million adults in the U.S. and costs an estimated $635 billion annually [4]. Moreover, chronic pain prevalence is expected to increase in the coming years, due to an aging population, increase in cases of diabetes and cancer survival rates [7]. Therefore, the search for new analgesic compounds that present a therapeutic alternative is crucial.

## 2. Plant Extracts and Pain Management

For over 7000 years, various extracts of plants have served as analgesics [8]. Indeed, morphine (the gold standard in analgesic therapy) is a plant alkaloid [8]. These plant extracts offer an opportunity to identify new analgesic compounds that may contain analgesic properties [9]. Identifying new compounds requires a strategy that combines analytical purification, pharmacological analyses and reliable resources [9]. As well, whole plant extracts may offer an advantage in approaching pain management from a polypharmacological standpoint because of their chemical complexity [10]. 

### 2.1. Corydalis yanhusuo W. T. Wang

#### 2.1.1. Botany and Traditional Uses

**Figure 1:**
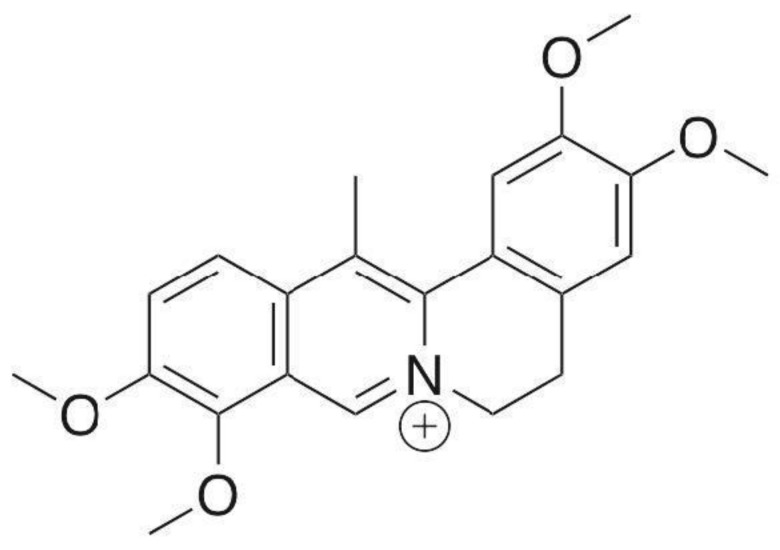
Chemical structure of DHC.

*Corydalis yanhusuo* W. T. Wang, known as Yanhusuo or Xuanhu, is a perennial herbaceous plant belonging to the Papaveraceae family, which is widely distributed in China, Japan, Korea, and other Asian countries [11]. The *C. yanhusuo* leaves can either be two-lobed or three-lobed and its racemes contain 5–15 sparse flowers [12]. The petals are colored purple or red; the male flowers have six stamens bundled into two filaments, and the female flowers have an oblate columnar ovary, a subcircular stigma; the fruit is a linear capsule [12]. The flowering season for *C. yanhusuo* is during April, while fruiting occurs from May to June. Figure 1 displays the flowering season of *C. yanhusuo.*

*Corydalis yanhusuo* is cultivated in the Zhejiang, Jiangxi, and Anhui provinces of China and has a long history of medicinal uses [12]. It was first recorded in Shennong Herbal Classic and was listed as a medium-grade drug [13]. *Corydalis yanhusuo* extracts (YHS) have been used for centuries as an analgesic agent in traditional Chinese medicine [13]. They were first documented in Lei Gong Pao Shi Lun between 618 and 907 AD and primarily used to alleviate chest pain [14]. 

There have been a few different accounts of the use of YHS through time. Hai Yao Ben Cao in the Tang Dynasty reported using it as a treatment for postpartum blood stasis [14]. Yi Xue Qi Yuan of the Jin and Yuan Dynasties reported using it to treat spleen and stomach stasis [15]. YHS has also been shown to alleviate pain caused by blood stasis, improve blood circulation, promote movement, and alleviate stagnation-induced pain [15]. Today, various studies have shown that YHS has pharmacological effects on the nervous, digestive, and cardiovascular systems, as well as therapeutic benefits in treating thrombosis and cancer [14,15]. The following sections will discuss YHS association with analgesia and inflammation, including its active components as well as what is known of its mechanism of action. 

#### 2.1.2. Most Recent Studies in Analgesia Induced by *Corydalis yanhusuo*

The extract of *C. yanhusuo* (YHS) has been studied for its role in analgesia over the years. However, a systematic study based on standardized animal pain assays was performed only recently by Wang et al. [16]. This study confirmed YHS analgesic effects in the tail flick, formalin paw licking, von Frey filament, and the hot box assays after spinal nerve ligation in mice. In this study, they demonstrated that YHS effectively attenuates acute, inflammatory, and neuropathic pain without causing any tolerance [16]. In addition to the various pain assays conducted, Wang et al. also showed that in dopamine D2KO mice, the antinociceptive effects of YHS were decreased in both the acute and neuropathic pain assays [16]. The study concluded that the effects on acute and neuropathic pain are mediated at least in part through the dopamine D2 receptor [16]. 

## 3. YHS Chemical Components

Thus far, there are more than 160 compounds that have been isolated from YHS; these include alkaloids, organic acids, volatile oils, amino acids, alcohols, and sugars [17]. Alkaloids are considered the most important biological active constituents of YHS [18]. More than 80 alkaloids have been isolated and identified from YHS; these include tertiary amines, quaternary alkaloids, and many non-alkaloids [19]. To date, however, there has been no comprehensive review on the phytochemical and the pharmacological effects of YHS in regards to its role in analgesia. Based on YHS high therapeutic value, we sought to systematically summarize the latest findings regarding its phytochemical and pharmacological effects and its bioactive components by using Google Scholar and the journal databases Scopus, PubMed, and CNKI (years 1982–2019). 

### 3.1. Alkaloids

The earliest study of YHS alkaloids was published in 1928 [20]. Alkaloids are the primary constituents of the YHS extract and play a crucial role in pain relief [20]. Thus far, a total of 80 alkaloids have been isolated from the plant; YHS contains about 0.5 to 1% of total alkaloids. Out of all the alkaloids, Dehydrocorydaline (DHC) is the most abundant alkaloid found in YHS and accounts for about 50% of the alkaloid content [21]. Next is corydaline (an analog of DHC), which is also present in high amounts [21]. The following sections will discuss various alkaloids and their relation to pain and inflammation. 

#### 3.1.1. Dehydrocorydaline (DHC)

Dehydrocorydaline is an alkaloid found in YHS (Figure 1). As mentioned before, DHC is the most predominant of the 80 alkaloids in YHS [21]. Studies have related DHC and its anti-inflammatory and anti-tumor effects [22]. 

Most recently, DHC was evaluated for its antinociceptive effects in in vivo mouse models [22]. One study looked at two inflammatory pain models (the acetic acid-induced writhing and formalin paw assays). It was found that the intraperitoneal administration of DHC at varying doses (3.6, 6, or 10 mg/kg) was able to attenuate pain in both assays [22]. It is worth mentioning that the locomotor or motor responses of these mice were not affected by DHC injections. To study the possible involvement of DHC with the opioid receptor, naloxone was found to reduce DHC antinociceptive effects, indicating that the DHC mechanism of analgesic action may involve the opioid system. [22]

Another study looked at the effects of DHC in bone cancer pain [23]. Previous studies have linked two functional polarization states of microglia, which activates M1 and M2 in the spinal cord during nerve injury induced neuropathic pain [23]. This study sought out to determine whether microglia in the spinal cord polarizes to M1 and M2, as well as if it contributes to the development of bone cancer [23]. The study used a mouse model with bone cancer to characterize this M1/M2 phenomenon, as well as investigate the antinociceptive effects of DHC during bone cancer development [23]. The study concluded that intraperitoneal administration of DHC (10 mg/kg) had antinociceptive effects on day 14 after osteosarcoma cell implantation [23]. In addition, they determined that there was a suppression of M1 phenotype and an upregulation of M2 phenotype of microglia within the spinal cord [23]. Furthermore, these results demonstrate that an imbalance in the polarization of microglia is towards the M1 phenotype in the spinal cord and this may contribute to the development of bone cancer pain [23]. As well, DHC may be a potential alkaloid to attenuate bone cancer pain. 

#### 3.1.2. Levo-Tetrahydropalmatine (l-THP)

**Figure 2:**
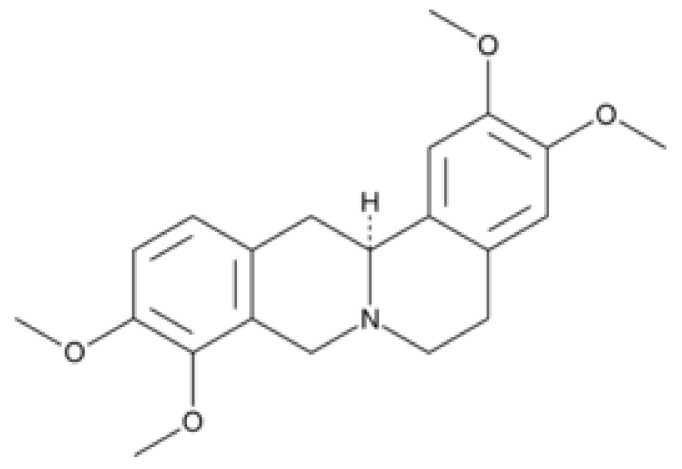
Chemical structure of l-THP.

Tetrahydropalmatine (THP) is an active ingredient of YH and has been used for the treatment of headaches or mild pain [24]. THP is also found in other plants, such as *Stephania rotunda* [24]; it contains two enantiomers. Figure 2 shows the chemical structure of Levo-tetrahydropalmatine (l-THP). L-THP is the more potent enantiomer and has been marketed worldwide under various brand names as an alternative analgesic [24]. L-THP has been shown to have analgesic effects and may be beneficial for the treatment of heart disease, as well as liver damage [25]. Furthermore, it has also been shown to be a potential in the treatment of drug addiction to cocaine and opiates [26]. 

The metabolism of l-THP involves its demethylation at several sites, with a number of demethylated metabolites identified in urine and feces [27]. A study led by Dr. Jin Guozhang from the Shanghai Medical Institute investigated the pharmacological profile of l-THP [26] and found that l-THP antagonizes the dopamine D1 and D2 receptors [26,27]. l-THP also binds to the dopamine D3 receptors, which is thought to be involved in preventing relapse [28]. 

As a traditional analgesic agent, l-THP has been used for the treatment of mild-to-moderate pain in China [24]. One study looked into the analgesic effect on chronic pain, as well as its mechanism. It showed that l-THP had an antihyperalgesic effect on neuropathic and inflammatory pain [24]. In addition, this study showed that a selective D1 receptor antagonist blocked the antihyperalgesic effect of l-THP, which further suggests that this action is mediated by activating D1 receptors [24]. Lastly, they indicated that l-THP exerts a significant antinociceptive effect in models of chronic inflammatory and neuropathic pain in mice without producing any motor deficits [24].

l-THP has also been studied as a potential agent in the treatment of addiction [26]. l-THP has been shown to attenuate cocaine self-administration in rats. One study indicated that l-THP has a therapeutic utility for the treatment of addiction [26]. The effects of l-THP on cocaine’s discriminative stimulus and reinforcing effects were said to involve likely dopamine (DA) receptors [26]. In addition, other studies suggest that l-THP binds to dopamine receptors [24]. One study assessed l-THP’s ability to block substitution of the D2/D3 agonist, (±) 7-OH-DPAT, for cocaine [26]. Based on previous reports, (±) 7-OH-DPAT dose-dependently substitutes for cocaine. Like cocaine, the dose–response curve for (±) 7-OH-DPAT was shifted to the right by l-THP pretreatment [26]. Just like the YHS extract itself, l-THP has sedative properties, which may limit its use in clinical settings. It should be noted that l-THP interacts with other receptors, such as alpha-1 adrenergic receptors and gamma-aminobutyric acid (GABA)_A_ receptors [27]. Even though l-THP has analgesic properties, it is not known whether it interacts with opioid receptors and whether its analgesic effects are naloxone-independent [28].

#### 3.1.3. Dehydrocorybulbine (DHCB)

**Figure 3:**
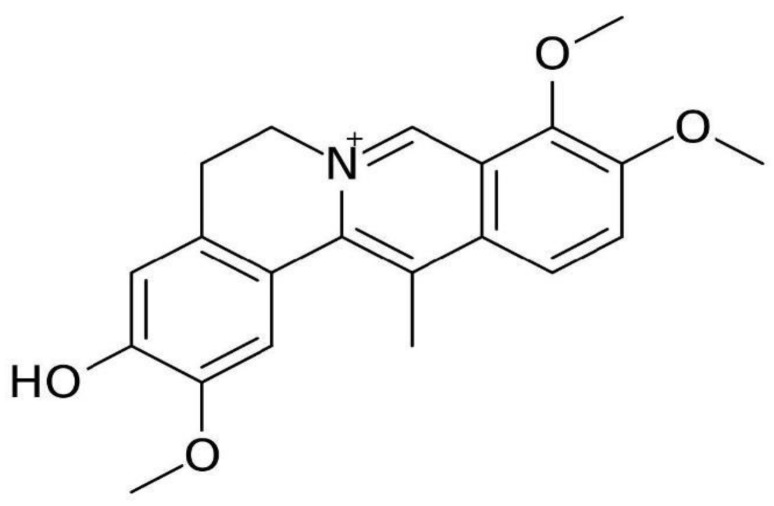
Chemical structure of DHCB.

Dehydrocorybulbine (DHCB) was originally isolated from *Corydalis ambigua var amurensis* in 1964 [29]. Figure 3 shows the chemical structure of DHCB. In vitro, DHCB was shown not metabolized in phase I, but is slowly metabolized into two glucuronidated products in phase II [29]. These findings demonstrate that DHCB is able to penetrate the blood–brain barrier and has favorable pharmacokinetic properties [17]. Zhang et al. showed that DHCB displays antinociceptive activity in mice in the tail flick assay at doses that are non-sedative [17]. In addition, DHCB was tested in the formalin assay to demonstrate its effects in acute and persistent inflammatory pain responses [17]. One study revealed that DHCB was effective at suppressing responses to chemically induced, inflammatory-derived and injury-induced pain [17]. This study also demonstrated no cause of antinociceptive tolerance [17]. In addition, this study examined YHS’s effect on the dopamine D2 receptor knockout mice and found that YHS’s antinociceptive effects are attenuated in acute and neuropathic pain but not inflammatory pain assays [17]. Lastly, they concluded that these effects are partially mediated through D2 receptor antagonism [17].

Another study looked at DHCB and its effect on neuropathic pain [28]. In this study, they evaluated the contribution of DHCB to P2X4 signaling in the modulation of pain-related behaviors and the levels of pronociceptive interleukins and proteins after spinal cord injury in rats [29]. They found that when DHCB was administered through IV, there was pain relief in the rat contusion injury models without disturbing motor function [29]. Furthermore, both P2XR and D2 receptor agonists antagonized DHCB’s antinociceptive effect, confirming other studies [17,29].

#### 3.1.4. Berberine

**Figure 4:**
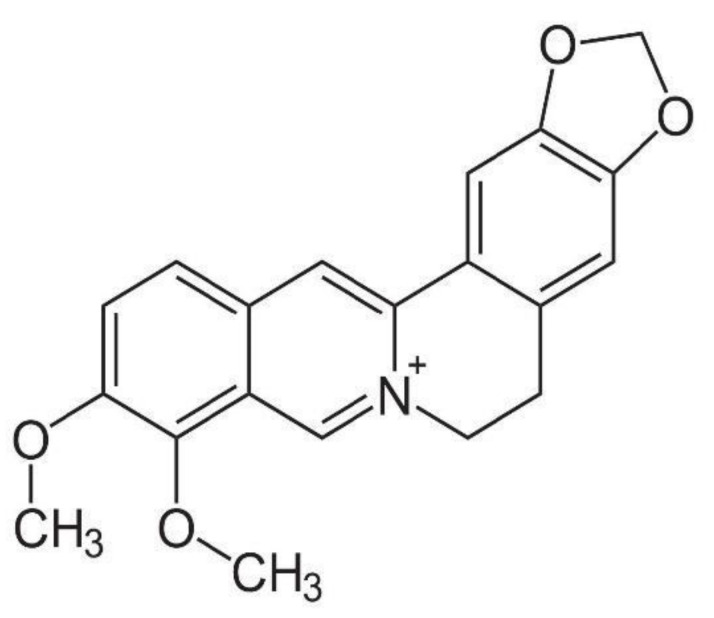
Chemical structure of berberine.

Berberine (shown in Figure 4) is a known active component of YHS that has been reported to play a prominent analgesic role in some pathological conditions of pain [30]. This compound belongs to the protoberberine alkaloid group, and it exists as an equilibrium mixture of three tautomeric forms [31]. Plants of the genus *Berberis* (~450–500 species) from the Berberidaceae family represent the main source of berberine and they have been used extensively to treat ailments in Asia for more than 3000 years [31]. Structural modifications have been made to improve berberine bioactivity, mainly focusing on substituents at the C-8 and C-13 positions to increase antimicrobial activity. Substituents at the C-9 position were also modified to increase anti-tumor activity [31]. Notably, the nitrogen at the conjugated molecular structure of berberine-related alkaloids was associated with cholesterol-lowering and anti-cancer effects [31]. To date, however, berberine is most known for its role in analgesia.

Berberine has been shown to display potent analgesic activity [32]. One study investigated berberine’s role in visceral pain, observing that berberine antinociceptive activity was blocked by the administration of the opioid receptor antagonist naloxone or by selective mu and delta morphine receptor (MOR and DOR) antagonists [33], which suggests that berberine binding activities at MOR and DOR may be responsible for visceral pain analgesia [32]. Interestingly, berberine also has an effect on the endogenous opioid system due to its ability to easily cross the blood–brain barrier [34]. After 7 days of berberine administration, MOR and DOR expression appeared to be significantly upregulated in cortical neurons [34]. Another study indicated that berberine alleviates neuropathic pain in a dose-dependent manner [35,36]. Using peripheral nerve injury in rats to induce neuropathic pain, berberine was observed to elevate mechanical and thermal thresholds with increasing paw withdrawal latency at increasing dosages [35,36]. Berberine administration also decreased TRPV1-mediated licking behaviors in rats, further suggesting that berberine reduces neuropathic pain through the TRPV1 mechanism [35,36].

It has also been proposed that the anti-cholinesterase activity of berberine is involved in its analgesic mechanism [37]. This is significant due to numerous studies reporting that intrathecal administration of acetylcholinesterase (AchE) inhibitors not only alleviated chronic pain but also increased the analgesic activity of intravenous alfentanil [37]. In an LPS-induced mouse model of neuroinflammation, berberine appeared to decrease oxidative stress, restore COX-2, and suppress AchE and inflammatory cytokines, such as NF-κB, TNF-α, and IL-6 [37].

#### 3.1.5. Palmatine

**Figure 5:**
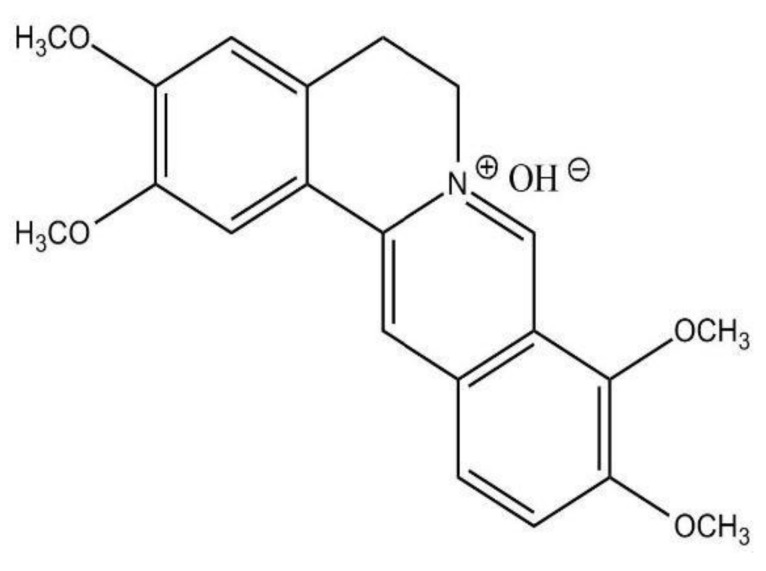
Chemical structure of palmatine.

Palmatine is an isoquinoline alkaloid from the class of protoberberine and a close structural analog of berberine [38]. Figure 5 shows the chemical structure of palmatine. It is found in traditional Chinese medicines, including *Tinospora cordifolia* (Willd.), *Coptis chinensis*, *Corydalis yanhusuo*, *Phellodendron amurense Rupr.*, *Tinospora sagittata* (Oliv.), Gagnep. and *Stephania yunnanensis H.S. Lo* [38]. Palmatine’s main metabolic pathways are demethylation and hydroxylation in phase I and glucuronidation and sulfation in phase II [39]. 

Palmatine has a variety of useful activities, particularly a role in analgesia and anti-inflammation [40]. It has been found that palmatine reduces the levels of proinflammatory cytokines IL-6 and TNF-α in LPS-induced murine macrophage-like cells and BALB/c mice [40]. Another study determined that palmatine inhibited TRIF-dependent NF-κB pathway, which causes a decrease in the production of proinflammatory factors and an increase in the production of anti-inflammatory factors [41]. Palmatine was also found to decrease the P2X7 receptor expression and phosphorylation of extracellular signal-regulated kinase 1 and 2 (ERK 1/2) in the hippocampus, which may relate to palmatine’s ability to diminish allodynia, and hyperalgesia in rats with concomitant diabetic neuropathic pain [41].

#### 3.1.6. Oxyacanthine

**Figure 6:**
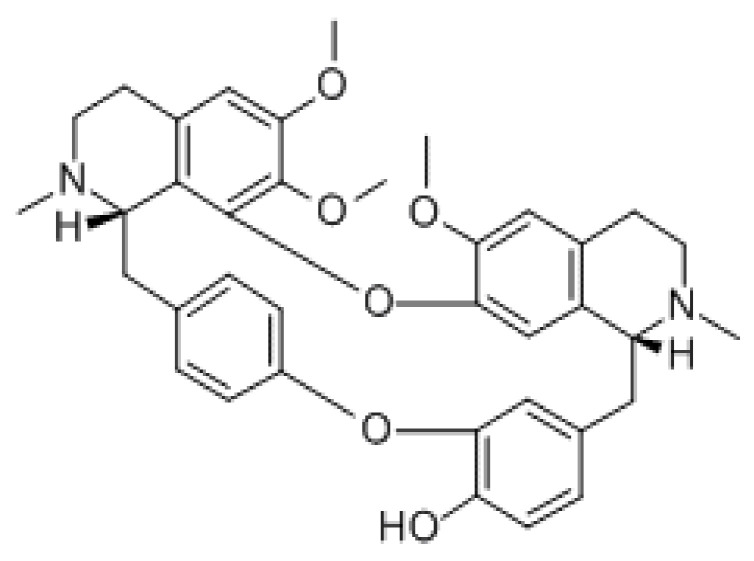
Chemical structure of oxyacanthine.

Oxyacanthine is a macrocycle, tertiary amino compound, a member of phenols, and a bisbenzylisoquinoline alkaloid (Figure 6) [42]. It can be isolated most notably from *Berberis vulgaris* roots [42]. Bisbenzylisoquinoline alkaloids have the potential to be anti-inflammatory drugs for their ability to prevent the synthesis or action of some proinflammatory cytokines [42]. Oxyacanthine has been shown to alleviate pain in vivo; however, not much is understood about how this compound works. One study suggests that oxyacanthine shows anti-inflammatory properties but is less effective than berberine by testing it on acute inflammation of paw edema in mice [42]. There have not been many studies involving oxyacanthine, so not much is known about the mechanism, pathway, or toxicity of the alkaloid. 

#### 3.1.7. Magnoflorine

**Figure 7:**
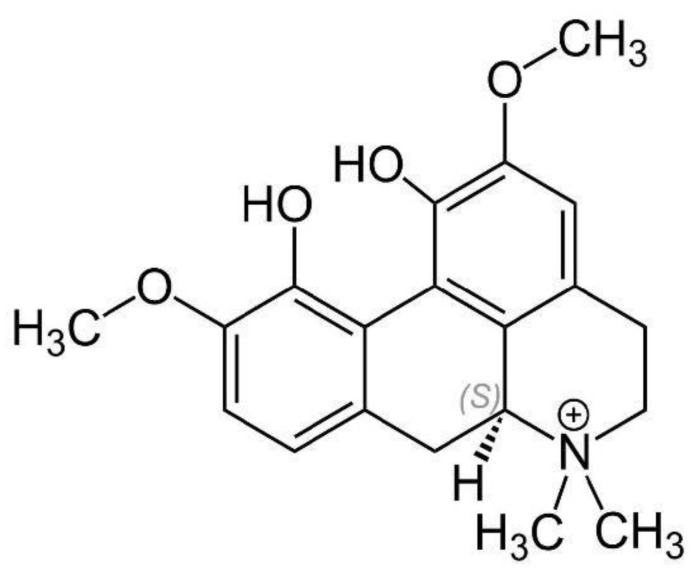
Chemical structure of magnoflorine.

Magnoflorine, also named thalictrine and escholine, is an isoquinoline alkaloid that has an aporphine configuration [43]. It is a quaternary ammonium alkaloid making it normally soluble in water, methanol, and ethanol and insoluble in low-polar organic solvents (e.g., petroleum ether and chloroform) (Figure 7) [43]. Magnoflorine has been reported to be found in herbal medicines, such as *Phellodendron amurense Rupr.*, *Sinomenium acutum (Thunb.) Rehder and E.H. Wilson*, *Thalictrum isopyroides C.A. Mey.*, *Magnolia officinalis Rehder & E.H. Wilson*, and *Berberis kansuensis C.K. Schneid.* [43]. Naturally, it is a secondary metabolite that is produced during the metabolic process of these plants and concentrated within the root, rhizome, stem, or bark [43]. 

Magnoflorine is of particular interest due to its structural similarity to morphine and possible link to analgesia [43]. One study demonstrated that it displays anti-inflammatory effects at high doses [44]. This study also showed that it could inhibit nitric oxide (NO) inflammation production and protect murine macrophage cells (RAW 264.7) from lipopolysaccharide-induced apoptosis [44]. Magnoflorine was also identified as a potential NF-κB inhibitor, which could negatively regulate the BRAF protein involved in the MAPK signaling pathway to inhibit the inflammatory response caused by the *Pseudomonas aeruginosa* PAK strain [45,46]. A recent study discovered that magnoflorine dose-dependently decreases the expression of several proinflammatory cytokines (TNF-α, IL-1β and IL-6) [47]. In turn, this determined that the possible mechanisms are related to the inhibition of Toll-like receptor 4-mediated NF-κB and MAPK signaling pathways [47]. Furthermore, these various findings indicate the potential use of magnoflorine to treat inflammation-related diseases. Current research suggests that magnoflorine is not toxic in most cells and may be a relatively safe molecule, although toxicity tests in vivo are still lacking [43].

Zhou et al., Tian et al. and Xue et al. conducted studies that have shown that magnoflorine had low bioavailability and high absorption and elimination rates [48,49,50]. In spite of this, these pharmacokinetic characteristics are concerning and may hinder magnoflorine’s development as a new drug. Some studies found that magnoflorine’s pharmacokinetic behavior differs significantly in herbal medicines [50]; this suggests that magnoflorine’s potential for treatment should be studied in combination with other drugs to improve its pharmacokinetic profile [50]. 

#### 3.1.8. Columbamine

**Figure 8:**
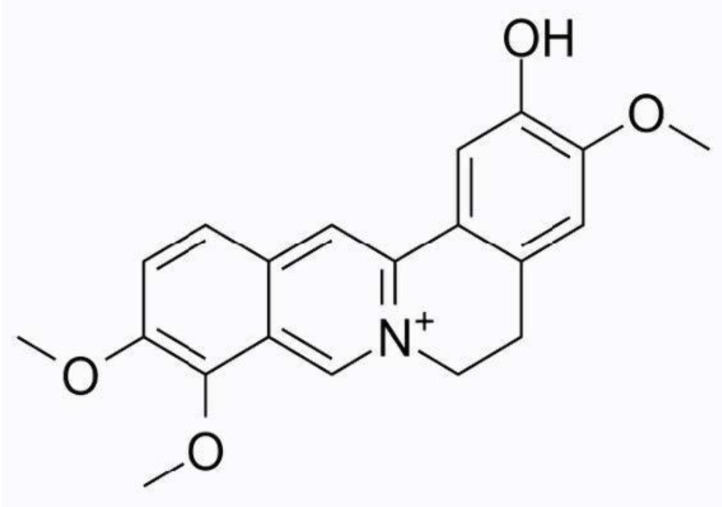
Chemical structure of columbamine.

Columbamine is a tetrahydroisoquinoline alkaloid belonging to the protoberberine group [51]. It has been identified in various plant species. It is an active component of Coptidis rhizoma, the dried rhizome of Coptis chinensis Franch., C. deltoidea C. Y. Cheng et Hsiao or C. teeta Wall., widely used in Traditional Chinese Medicine [52]. Figure 8 illustrates the chemical structure of columbamine. 

Columbamine has been shown to relieve pain and inflammation. Recent studies have demonstrated columbamine inhibits monoamine oxidase B (MAO-B), which is a crucial enzyme that deaminates dopamine [51]. Due to its involvement in dopamine metabolism, MAO-B inhibitors have exhibited antinociceptive activity in neuropathic pain and postoperative pain [51]. In addition, binding models of columbamine with MAO-B active sites revealed that it buries itself inside the pocket of MAO-B, a mechanism similar to safinamide inhibition [51]. Through a MAO-B enzyme activity assay, columbamine showed a moderate inhibitory effect by decreasing MAO-B enzyme activity by 40% [52]. As a result, this may be essential in explaining columbamine’s partial involvement in nociception within the central nervous system [51]. 

In another study led by Liu et al, the anti-inflammatory and antinociceptive properties of columbamine were evaluated using xylene-induced ear edema and acetic acid-induced writhing mouse models, respectively [53]. They concluded that columbamine significantly inhibited both the xylene-induced ear edema and abdominal contortions at a higher dosage of 56 mL/kg [53]. There is no clear indication of columbamine’s mechanism of action. 

#### 3.1.9. Corydine and Corydaline

**Figure 9:**
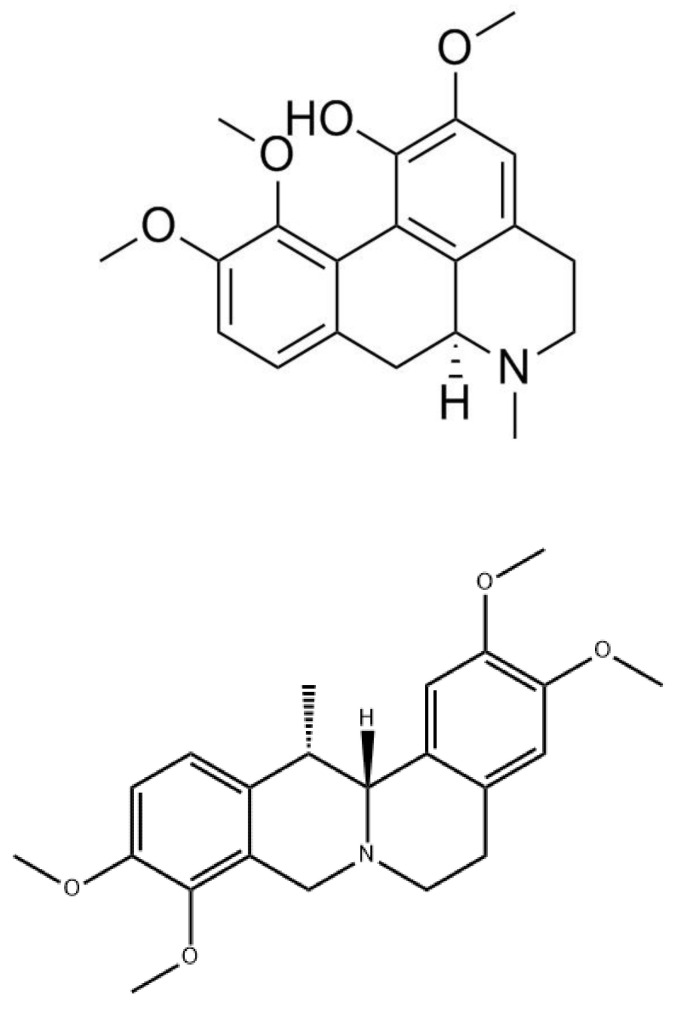
Chemical structure of corydine (**top**) and corydaline (**bottom**).

Corydine (top) and corydaline (bottom) are two isoquinoline alkaloids naturally occurring in many species of corydalis [54]. Figure 9 shows the chemical structure of both compounds. Many studies have evaluated the effect of corydine and corydaline in analgesia through various pharmacophore-based virtual (screening and docking) and pharmacological (in vitro binding and functional assays, and behavioral tests) approaches [54]. One study reported that these two alkaloids are new MOR agonists that produce antinociceptive effects in mice after subcutaneous administration via a MOR-dependent mechanism [54]. Based on this, these two compounds were characterized as G protein-biased agonists to the MOR without inducing β-arrestin-2 recruitment upon receptor activation [54]. Thus, they may be viewed as novel opioid analgesics.

In the same study, the MOR agonist activity of corydine and corydaline were tested in vivo in a mouse model of chemical sensitivity, the writhing assay, which is a widely used model to illustrate visceral pain [54]. Corydine and corydaline displayed antinociceptive effects in mice by significantly inhibiting the writhing behavior [54]. When corydine and corydaline were administered, they significantly reduced the number of writhes by 51% and 59% [54]. In addition, the anti-writhing response was antagonized by the MOR antagonist naltrexone [54]. Collectively, this study shows that this response may be through a MOR-mediate mechanism of action [54].

Although corydine and corydaline were reported to produce antinociceptive effects in rodents, the mechanism of action has not been fully determined [54]. In spite of this, there needs to be more pharmacokinetic studies to investigate if corydaline can effectively cross the blood–brain barrier [55]. One study reported that O-demethylation and hydroxylation are the major corydine and corydaline metabolic pathways in the human liver [55]. In addition, some in vitro assays concluded that corydaline binds to the dopamine D1 receptor with some antagonist activity [55,56], but corydine was not tested [56].

## 4. Conclusions

For centuries, the YHS extract has historically been used to improve blood circulation, relieve pain caused by blood stasis, and treat a variety of diseases. Modern pharmacological studies have concentrated on the analgesic mechanisms of YHS, yet there are few comprehensive reviews of its isoquinoline alkaloids involved in antinociceptive activities. In this article, we reviewed the phytochemical and pharmacological effects of these alkaloids that have significant ties to analgesia.

## Data Availability

Not applicable.
